# Longitudinal genome‐wide aneuploidy measurements in circulating cell‐free DNA to predict lack of benefit from pembrolizumab in patients with metastatic urothelial cancer

**DOI:** 10.1002/1878-0261.70272

**Published:** 2026-06-16

**Authors:** Youssra Salhi, Stavros Makrodimitris, Sandra van Wilpe, Iris te Paske, Vanja de Weerd, Corine M. Beaufort, Hans M. Westgeest, Jens Voortman, Maureen J. B. Aarts, Maud Rijnders, Astrid A. M. van der Veldt, Ronald de Wit, Niven Mehra, Saskia M. Wilting, Debbie G. J. Robbrecht

**Affiliations:** ^1^ Department of Medical Oncology Erasmus MC Cancer Institute Rotterdam The Netherlands; ^2^ Department of Medical Oncology Radboud University Medical Center Nijmegen The Netherlands; ^3^ Department of Medical Oncology Amphia Hospital Breda The Netherlands; ^4^ Department of Medical Oncology Cancer Center Amsterdam, Amsterdam UMC, Vrije Universiteit Amsterdam The Netherlands; ^5^ Department of Medical Oncology, GROW Research Institute for Oncology and Reproduction Maastricht University Medical Center+ The Netherlands; ^6^ Department of Internal Medicine Franciscus Gasthuis & Vlietland Rotterdam The Netherlands; ^7^ Department of Radiology and Nuclear Medicine Erasmus MC Cancer Institute Rotterdam The Netherlands

**Keywords:** circulating tumor DNA, metastatic urothelial cancer, modified fast aneuploidy screening test‐sequencing system, pembrolizumab

## Abstract

Accurate prediction of lack of benefit from pembrolizumab in patients with metastatic urothelial cancer (mUC) is an unmet need. We investigated the dynamics of circulating tumor DNA (ctDNA) load, estimated using the modified fast aneuploidy screening test‐sequencing system (mFast‐SeqS), as a potential biomarker for early on‐treatment identification of treatment response. A total of 104 patients with mUC treated with pembrolizumab from two prospective biomarker discovery trials were included and mFast‐SeqS was performed on paired blood samples collected at baseline and on‐treatment. Patients with a high on‐treatment aneuploidy score (≥ 5, *n* = 26) had a shorter median OS than patients with a low (< 5) score (*n* = 76) (3 vs 17 months: *P*‐value< 0.001). Patients with an increased (*n* = 10), stable (*n* = 66), or decreased (*n* = 28) on‐treatment score relative to their baseline score had a median PFS of 1.5, 4.0, and 8.3 months, respectively. Median OS was 3.0, 11.1, and 18.7 months, respectively. In patients with mUC treated with pembrolizumab, the on‐treatment mFast‐SeqS‐based ctDNA level and its dynamics relative to baseline are independent prognostic markers that can be used to identify patients that are unlikely to benefit from pembrolizumab.

## Graphical abstract

Many patients with urothelial cancer do not benefit from treatment with pembrolizumab, while at risk of severe side effects. Changes in the levels of circulating tumor DNA early during treatment, measured by a simple and affordable assay that can be easily implemented in the clinic, can be used as a prognostic tool to identify these patients.

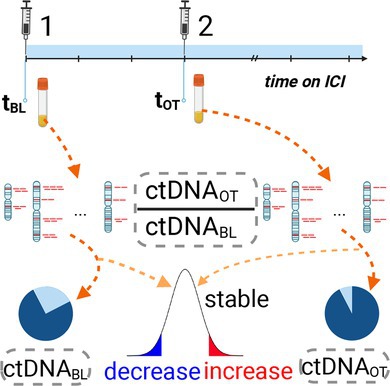

**Abbreviations**
AUC: area under the curve
cfDNA: cell‐free DNA
ctDNA: circulating tumor DNA
EDTA: Ethylenediaminetetraacetic acid
EVP: enfortumab vedotin and pembrolizumab
ICI: immune checkpoint inhibitor
L1: line1 element
mFAST‐SeqS: modified fast aneuploidy screening tool—sequencing system
mUC: metastatic urothelial cancer
OS: overall survival
PCR: polymerase chain reaction
PFS: progression‐free survival
RECIST: response evaluation criteria in solid tumors
ROC: receiver operating characteristic
SVM: support vector machine
UC: urothelial cancer

## 1 Introduction

Immune checkpoint inhibitors (ICIs) have improved survival in patients with metastatic urothelial cancer (mUC) [1, 2, 3]. However, since the first approvals, there has been an urgent need for biomarkers that could guide treatment decisions. Although durable responses are achieved even beyond 5 years, most patients develop progressive disease within the first 6 months of therapy [1, 4]. Recently, the EV‐302 trial showed that enfortumab vedotin, an antibody‐drug conjugate directed against NECTIN‐4, combined with pembrolizumab (EVP), resulted in doubling of the survival outcomes compared to standard of care platinum‐based chemotherapy in 1 L setting for patients with advanced UC [5]. However, 50% of the patients treated with EVP developed progressive disease within 13 months [5].

Consequently, accounting for ICIs as monotherapy and in a combination therapy such as EVP, many patients are exposed to therapies without deriving benefit but with the risk of potential severe toxicity. Not to mention the burden on the healthcare system. Therefore, there is an urgent need for easy‐to‐use biomarkers that can guide individualized treatment decisions.

With the development of PD‐(L)1 targeting ICI, PD‐L1 assays have been developed in parallel, and, despite limitations, these are still used as a biomarker [6, 7, 8, 9]. Biomarker research has been focusing on microsatellite instability or mismatch repair deficiency [10], tumor mutational burden [11, 12], and the tumor microenvironment [11, 13], all using tissue‐based approaches. Notwithstanding promising results, the representative value when using archival tissue is debatable, new tissue biopsies are an additional burden to the patient and not always obtainable, and tissue samples may not represent the full disease heterogeneity. Liquid biopsies, that is, the detection of tumor‐specific signals in patients' body fluids, can overcome these drawbacks, but bring other challenges. Impressive results have been obtained in both locally advanced and metastatic UC using highly sensitive, tumor‐informed, personalized ctDNA assays [14, 15]. However, this approach, in which patient‐specific ctDNA detection panels are designed based on sequencing of tumor tissue, is rather complicated, which hampers worldwide clinical implementation. Tolmeijer et al combined a targeted panel with tissue‐based information and high amounts of cfDNA to optimize detection sensitivity and showed that early on‐treatment dynamics are prognostic of progression under ICI [16].

Tumor‐agnostic approaches aimed at the detection of universal cancer‐specific features, such as chromosomal instability, would greatly facilitate implementation in daily clinical practice but, in general, exhibit more limited sensitivities.

Here, we investigate whether the previously described mFast‐SeqS method, a tumor‐agnostic and affordable assay for the detection of tumor‐specific aneuploidy in the blood [17], can be used to monitor treatment response early during treatment. This assay has limited sensitivity, but high specificity and can thus identify patients with high levels of ctDNA. In addition, the short hands‐on time and low input DNA requirements (1 ng) [17] render it promising for broader adoption. Recent work in breast cancer demonstrated that the assay could inform clinically relevant decisions regarding treatment selection [18].

Previously, we showed a clear association between the mFast‐SeqS‐based aneuploidy score in cfDNA before start of treatment and response to ICI pembrolizumab in patients with advanced UC [19]. The genome‐wide aneuploidy score (≥5) in cfDNA can be considered as a proxy for the levels of circulating tumor DNA (ctDNA) and a high score prior to the initiation of pembrolizumab identified patients who were unlikely to have benefit from pembrolizumab. However, a low aneuploidy score at baseline was not associated with response, resulting in high specificity but limited sensitivity [19].

To further explore the potential of the mFast‐SeqS assay in assessing response to ICIs in mUC patients, we analyzed the relation between clinical outcomes and longitudinal aneuploidy scores measured prior to (baseline, t_BL_) and following 1 or 2 cycles of pembrolizumab (on‐treatment, t_OT_). To evaluate whether the mFast‐SeqS based aneuploidy score could be supportive for decision‐making on (dis)continuation of pembrolizumab, we have built machine learning models containing both clinical and ctDNA‐based parameters to predict the patient‐specific risk of early progression.

## 2 Materials and methods

### 2.1 Patients and outcomes

Patients with locally advanced or mUC from the prospective phase II Dutch RESPONDER (*n* = 84, enrolled between September 2017 and July 2020; approved by Foundation BEBO ‐ Evaluation of Ethics in Biomedical Research—Assen, the Netherlands, NL61719.056.17, NCT03263039) and the prospective Dutch PRECISE biomarker discovery trials (*n* = 60, enrolled between March 2017 and August 2022; approved by Radboud University Medical Center medical ethics committee, dossier number NL60249.091.16) were initially included. We excluded 40 patients: 15 because they did not start with pembrolizumab, 1 due to the presence of a second primary tumor, 21 due to the absence of an on‐treatment sample after one or two cycles of pembrolizumab, 2 that had progressive disease before the on‐treatment sample was taken, and 1 because of missing survival information, leaving 104 patients in the current analysis (RESPONDER, *n* = 58; PRECISE, *n* = 46). Both trials were conducted in accordance with the Declaration of Helsinki and approved by the independent local medical ethical committees. All patients provided written informed consent and were treated with pembrolizumab in first‐ or second‐line setting following a three‐week schedule (200 mg fixed dose q3w) until progressive disease. Treatment response was determined according to the response evaluation criteria in solid tumors (RECIST) v.1.1 every 12 weeks using computed tomography. The following clinical parameters were additionally collected: gender, age at inclusion, WHO performance score at inclusion, the presence of visceral metastases, Progression‐Free Survival (PFS), and Overall Survival (OS).

### 2.2 Plasma analysis

In the RESPONDER trial, blood was collected prior to start and after one or two cycles of pembrolizumab in blood‐stabilizing CellSave tubes and processed to plasma within 96 h as described in [20]. cfDNA was isolated using the QIAamp® Circulating Nucleic Acid kit (QIAGEN, Venlo, the Netherlands) according to the manufacturer's instructions from an average of 1726.7 μL plasma (range: 350–2000 μL), on average yielding 88.5 ng cfDNA (range: 13.132–844.2 ng) as measured on the Qubit fluorometer (dsDNA High Sensitivity assay by Thermo Fisher Scientific, Waltham, MA). In the PRECISE study, blood was collected in EDTA or cfDNA collection tubes (Roche tubes). EDTA and cfDNA tubes were processed as previously described by Tolmeijer et al. [16].

### 2.3 mFast‐SeqS assay

To assess aneuploidy in cfDNA, the mFast‐SeqS approach was essentially performed as described by Belic et al. [17]. Line‐1 (L1) amplicon libraries were prepared from 1 ng cfDNA using target‐specific L1 primers and Phusion Hot Start II Polymerase. To increase complexity of the resulting sequencing libraries, a random spacer was introduced to the forward primer as described before [21]. The resulting PCR products were purified by AMPure Beads (Beckman Coulter) and used for a second PCR, in which sequencing adaptors and sample‐specific indexes were added. L1 amplicon libraries were pooled and sequenced on a MiSeq (Illumina) generating at least 90 000 single‐end reads of 150 bp.

The obtained reads were mapped to the hg19 genome with Burrows–Wheeler alignment, version 0.7.4 [22]. The number of reads originating from each chromosome arm was divided by the total number of reads generated in each sample and compared to corresponding numbers observed in cancer‐free controls of the same gender (18 females and 17 males) as follows: A Z‐score was calculated per chromosome arm by subtracting the mean and dividing by the standard deviation of the coverage‐corrected read counts in control samples to test for over‐ and underrepresentation of each chromosome arm. The short arms of chromosomes 13, 14, 15, 21, and 22, as well as chromosome Y, were excluded due to the insufficient presence of LINE‐1 elements. The resulting Z‐scores per chromosome arm were squared and summed into a genome‐wide aneuploidy score per patient. Patients with a genome‐wide aneuploidy score ≥ 5 were defined as ctDNA‐high and those with < 5 as ctDNA‐low based on the cut‐off described by Belic et al., which has been validated in various types of solid tumors [17].

### 2.4 Dynamics of the aneuploidy score

To investigate dynamics in the aneuploidy scores, we first calculated the log‐transformed ratio of the sum of squared *Z*‐scores of the on‐treatment sample to the baseline sum of squares. We then divided the cohort into three subgroups; patients with a ctDNA increase, patients with a ctDNA decrease and patients with a stable ctDNA level during treatment. This was determined based on whether a patient's follow‐up sample had significantly higher or lower aneuploidy score compared to what one would expect according to the assay's technical variation. To ascertain the latter, we leveraged 37 independent samples not included in this cohort for which we ran two technical replicates (Fig. S1). Assuming that the sign of the difference between replicates does not matter and that the variability is independent of the patient's aneuploidy score, we modeled the absolute log‐ratio between the sum of squares of a pair of replicates using a half‐normal distribution. The 95th percentile of this distribution was used as a cutoff, that is, patients whose sum of squares increased (decreased) more than this cutoff were assigned to the ctDNA increase (ctDNA decrease) group, while patients whose change in aneuploidy score was within this 95% window were deemed as ctDNA stable.

We used the aneuploidy score dynamics in Cox proportional hazards models as an ordinal variable (ctDNA‐decrease, −stable, and –increase), which we implemented via a ‘staircase encoding’: The ctDNA‐decrease group was included in the model's intercept and two binary dummy variables were introduced to model the effect of ctDNA‐stable vs ctDNA‐decrease and ctDNA‐increase vs ctDNA‐stable (Table S1). Based on this encoding, the hazard ratio between the ctDNA‐increase and ctDNA‐decrease groups can be calculated by multiplying the hazard ratios calculated for the two dummy variables.

### 2.5 Statistical analyses

Survival curves were estimated using the Kaplan–Meier method, and differences between groups in survival analyses were assessed using the log‐rank test. For continuous and ordinal predictors, as well as for multivariable survival regression, we employed Cox proportional hazards models, using Efron's method to handle ties. To test whether the prognostic value of the aneuploidy score is different between patients receiving pembrolizumab in the first or second line, we also trained two additional models including (a) an interaction term between the treatment line and the baseline aneuploidy score and (b) an interaction term between the treatment line and the (continuous) aneuploidy ratio. All analyses were performed using either SPSS (v.25), R (v3.6.1.) (R Core Team, 2017), or Python 3.11 as needed. *P*‐values < 0.05 were considered significant.

We investigated whether the aneuploidy scores were associated with clinical variables (tumor mutational burden, PD‐L1 status) and with the inclusion center. To this end, we used mixed linear models to predict the log‐transformed aneuploidy score with a random intercept per patient and fixed effects for time and the variable(s) of interest. These analyses were performed on the subsets of patients without missing data.

### 2.6 Prediction of PFS based on baseline and dynamics of the aneuploidy score

To test whether the aneuploidy score and its dynamics can be used to guide treatment decisions regarding lack of response to pembrolizumab, we combined these data with clinical parameters and built survival models to assess the risk of progression. We divided the parameters into three sets (clinical parameters, baseline aneuploidy score—t_BL_—and on‐treatment aneuploidy score—t_OT_) and compared all seven combinations of these three sets (Table S2) using four different models: survival Support Vector Machine (SVM) with linear kernel, Cox regression with elastic net regularization, survival tree, and survival forest.

To select the best classifier, hyperparameters, and feature set, we employed threefold cross‐validation stratified by inclusion center and a grid search over the models' hyperparameters, which are listed in Table S3. The mean ROCAUC for identifying patients with progressive disease at 6 months, across the folds was used as criterion to select the best combination.

To obtain an unbiased estimate of the future performance of our model on unseen data, we adapted the procedure above as follows: The dataset was divided into a training and a test set of equal size stratified by both inclusion center and progressive disease within six months or not. In the training set, we found the optimal combination of feature set, classifier, and classifier hyperparameters using threefold cross‐validation stratified by inclusion center. The optimal model as determined by the cross‐validation was then trained on the entire training set and evaluated on the unseen test set in terms of the concordance index and the ROC AUC at 6 months. This procedure was repeated 200 times, each time making a different training/test split to provide us with more robust estimates.

We then tested the potential of our best model in guiding treatment decisions regarding treatment discontinuation by decision curve analysis [23]. For each patient, we obtained the model's estimate for the probability that the patient will have progressive disease within 6 months from the initiation of pembrolizumab using 10‐fold cross‐validation. The nature of this decision (i.e., to stop with ICI if the model predicts that a patient will progress quickly) calls for conservativeness to avoid undertreating patients who would benefit from ICI. We thus set the range clinically relevant probability thresholds from 0.6 to 0.9 and compared our model's net benefit to that of the standard strategies, namely ‘intervene for none’ (never stop treatment early) and ‘intervene for all’ (always stop treatment early) [23]. To also compare the added value of our model to existing biomarkers, we repeated this analysis on the 88 patients for whom PD‐L1 status was also known. In this experiment, we also compared our model's net benefit to that of stopping if a patient is PD‐L1 negative.

## 3 Results

### 3.1 Baseline characteristics of the total cohort

**Fig. 1:**
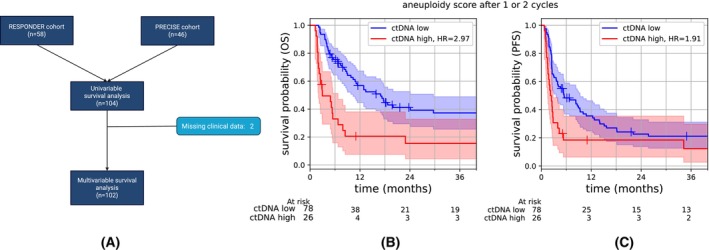
Study description and on‐treatment survival analyses. (A) Description of the cohort and number of included patients. (B) Kaplan–Meier curves for overall survival (OS) of patients with a high (red) and low (blue) aneuploidy score while on‐treatment (t_OT_). Shaded areas denote 95% confidence intervals. (C) As in (B), but for progression‐free survival (PFS). Statistical significance was assessed using the log‐rank test.

All patients who participated in the RESPONDER (*n* = 58) and PRECISE (*n* = 46) trials with baseline and on‐treatment samples for ctDNA analyses were included for univariable analyses, leading to a combined cohort of 104 patients (Table 1). Of them, 75 patients received pembrolizumab in second line (72%), and 29 in first line. Median follow‐up was 53.3 months for PFS (IQR 40.3–59.5) and 51.9 (IQR 18.3–59.5) months for OS. For multivariable analyses, two patients were excluded due to missing clinical data (Fig. 1A). Median OS was 5.5 months for ctDNA‐high patients at t_BL_ vs 15.8 for ctDNA‐low (*P*‐value = 0.068), while median PFS was 2.6 vs 5.3 months (*P*‐value = 0.26, Fig. S2).

**Table 1:** Baseline characteristics of patients included for mFast‐SeqS analysis stratified by inclusion center.

Characteristic	RESPONDER cohort (*n* = 58)	PRECISE cohort (*n* = 46)	Total cohort (*n* = 104)	*P*‐value
Age, median [IQR]				0.14
	69 [64.25, 72.75]	71 [62.5, 76.75]	70 [63.75, 75]	
Gender, *n* (%)				0.36
Male	41 (71%)	37 (80%)	78 (77%)	
Female	17 (29%)	9 (20%)	26 (23%)	
Prior systemic treatment metastatic disease, *n* (%)				0.15
None	11 (19%)	18 (39%)	29 (28%)	
Carboplatin‐gemcitabine	22 (38%)	15 (33%)	37 (36%)	
Cisplatin‐gemcitabine	22 (38%)	9 (20%)	31 (29%)	
ddMVAC	3 (5%)	2 (4%)	5 (5%)	
Other*	0 (0%)	2 (4%)	2 (2%)	
Visceral metastases, *n* (%)				0.55
No	31 (53%)	27 (59%)	58 (55%)	
Yes	27 (47%)	18 (39%)	45 (45%)	
Unknown	0 (0%)	1 (2%)	1 (1%)	
WHO‐status, *n* (%)				0.005
0	17 (29%)	3 (7%)	20 (19%)	
1 or 2	41 (71%)	42 (93%)	83 (80%)	
Unknown	0 (0%)	1 (2%)	1 (1%)	

* One patient received carboplatin‐gemcitabine followed by paclitaxel and another docetaxel followed by carboplatin‐gemcitabine.

### 3.2 On‐treatment aneuploidy score is associated with clinical outcome

The on‐treatment samples were collected after one (*n* = 94) or two cycles (*n* = 10) of pembrolizumab, with a median of 21 days after the start of the treatment. Of those, 26 patients had a high aneuploidy score at t_OT_, while there was no significant association between number of treatment cycles (1–2) and high or low on treatment aneuploidy score (*P* = 0.71, Fisher's exact test). The aneuploidy scores were not associated with PD‐L1 status, tumor mutational burden, or inclusion center (Tables S4–S6).

Median overall survival (OS) was 3 months in patients with a high aneuploidy score compared to 17 months in patients with a low aneuploidy score at t_OT_ (*P*‐value < 0.001, log‐rank test, Fig. 1B). Similarly, patients with a high aneuploidy score had a significantly shorter PFS: median of 2.2 vs 5.5 months respectively (*P*‐value = 0.008, log‐rank test, Fig. 1C).

### 3.3 On treatment ctDNA change and clinical outcome

**Fig. 2:**
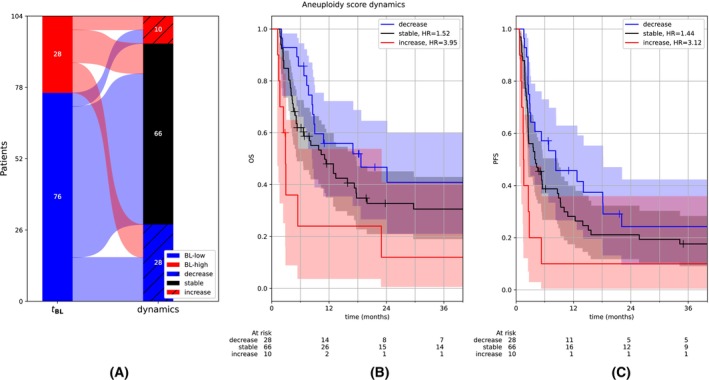
Circulating tumor DNA (ctDNA) dynamics. (A) Distribution of patients into the ctDNA dynamics groups (decrease/stable/increase‐right) based on their baseline (t_BL_) ctDNA levels (high/low‐left). (B) Kaplan–Meier curves for overall survival of patients with an aneuploidy score increase (red), stable (black) and decrease (blue) aneuploidy score after the point of follow‐up compared to baseline. Shaded areas denote 95% confidence intervals. (C) As in (b), but for progression‐free survival (PFS). Statistical significance was assessed using an ordinal Cox proportional hazards model.

We then studied the dynamics of the aneuploidy score. Due to the short time interval between blood draws, aneuploidy profiles showed little variation between the two samples from the same patient (Fig. S3). Thus, an increase (or decrease) in the aneuploidy score can be directly interpreted as an increase (or decrease, respectively) in ctDNA load. Based on our analyses of 37 pairs of independent technical replicates (Methods), we defined a log ratio of ± 0.56 to denote a significant deviation in t_OT_ ctDNA levels compared to t_BL_ beyond what is expected based on technical variation (Fig. S1). Following this definition, 63.5% (*n* = 66) of patients were deemed to be ctDNA‐stable. In 9.6% (*n* = 10) and 26.9% (*n* = 28) of patients, we observed a significant increase or decrease in ctDNA amounts, respectively. Most patients that were ctDNA‐low at t_BL_ (93.5%) either remained stable or decreased, while ctDNA‐high patients were more evenly distributed among the three groups (Fig. 2A).

Median OS was 3 months for the ctDNA‐increase group, 11.1 months for the ctDNA‐stable group, and 18.7 months for the ctDNA‐decrease group (Fig. 2B). Ordinal Cox proportional hazards modeling (Table S1) of OS showed that the ctDNA‐stable group had a hazard ratio of 1.52 compared to the ctDNA‐decrease group (95% CI [0.84, 2.74], *P*‐value = 0.17), while the ctDNA‐increase group had a hazard ratio of 2.60 (95% CI [1.26, 5.37], *P*‐value = 0.009) compared to the ctDNA‐stable group.

Similarly, patients with a ctDNA increase had a shorter median PFS compared to patients with a ctDNA stable score or a ctDNA decrease: 1.5, 4.0 and 8.3 months respectively (Fig. 2C), with a HR of 1.44 (95% CI [0.86, 2.41], *P*‐value = 0.16) for ctDNA‐stable patients compared to patients who showed a decreased aneuploidy score, while the ctDNA‐increase group had a hazard ratio of 2.17 (95% CI [1.06, 4.41], *P*‐value = 0.033) compared to the stable group.

The independent value of the aneuploidy score and its dynamics were subsequently evaluated in a multivariable analysis. We found that previous treatment for metastatic disease, study (RESPONDER vs. PRECISE), and t_BL_ aneuploidy score were significant prognostic factors for OS (Table 2). Conditioned on these variables, the ctDNA‐stable group had a significantly shorter OS than the ctDNA‐decrease group (HR = 2.26, *P* = 0.001, Table 2). There was no difference in OS between the ctDNA‐increase group and ctDNA‐stable group (HR = 1.49, 95% CI [0.64, 3.47], Table 2). The addition of the dynamics measurements to the clinical variables and t_BL_ ctDNA led to a statistically significantly better fit of the data (*P*‐value = 0.009, log‐likelihood ratio test with 2 degrees of freedom).

**Table 2:** Multivariable analyses for prognostic value of the baseline aneuploidy score and its dynamics over the first treatment course in terms of OS (left) and PFS (right). The hazard ratio (HR), 95% confidence interval around the HR estimate (CI), and the associated *p*‐value are shown for each variable. The presence/absence of visceral metastasis violated the proportional hazards assumption (i.e. demonstrated a time‐dependent hazard ratio), so we included it as a random effect and did not estimate its hazard.

	OS			PFS		
	HR	CI	*P*‐value	HR	CI	*P*‐value
Pembrolizumab in 2nd line	2.20	[1.18, 4.09]	0.013	3.39	[1.85, 6.19]	<0.001
Sex (ref. Female)	0.68	[0.38, 1.20]	0.19	0.60	[0.35, 1.03]	0.06
Study (ref. PRECISE)	2.09	[1.19, 3.69]	0.01	1.35	[0.81, 2.30]	0.26
WHO status > 0	1.68	[0.83, 3.40]	0.15	1.23	[0.67, 2.25]	0.51
Aneuploidy ≥ 5 at t_BL_	2.26	[1.21, 4.24]	0.011	1.52	[0.84, 2.74]	0.16
ctDNA‐stable vs ctDNA‐decrease	2.26	[1.18, 4.33]	0.014	1.90	[1.06, 3.40]	0.031
ctDNA‐increase vs ctDNA‐stable	1.49	[0.64, 3.47]	0.35	1.79	[0.78, 4.13]	0.17

Focusing on PFS, previous treatment and ctDNA‐stable vs ctDNA‐decrease were statistically significant variables (Table 2). After including the ordinal dynamics data, the multivariable model's fit significantly improved (*P*‐value = 0.016, log‐likelihood ratio test with 2 degrees of freedom).

Including the aneuploidy ratio as a continuous variable also led to similar conclusions (Table S7), confirming that ctDNA dynamics is an independent prognostic marker for both OS and PFS, while the t_BL_ ctDNA level was only significantly associated with OS. When measuring the association of ctDNA dynamics and response to pembrolizumab (defined as stable disease, partial response, or complete response after 6 months), the results were highly concordant with those for PFS (Table S8). We also found a statistically significant interaction between the aneuploidy dynamics and receiving pembrolizumab in the second line for PFS, but not OS (Table S9). This implies that cfDNA dynamics might carry more prognostic value for PFS in second‐line patients.

PD‐L1 status was only available for a subset of patients in our cohort. Including PD‐L1 status in the multivariable analysis of these patients led to similar results (Table S10), implying that ctDNA dynamics also have added value compared to PD‐L1 status alone.

### 3.4 Predicting risk of progression by machine learning

To evaluate whether we could use the available clinical and ctDNA‐based information to quantify risk of progression at t_BL_ and/or t_OT_, we employed machine learning. We compared different linear and nonlinear survival models (Table S3) and combinations of features (Table S2) in terms of the time‐dependent ROCAUC at 6 months from initiation of pembrolizumab treatment.

**Fig. 3:**
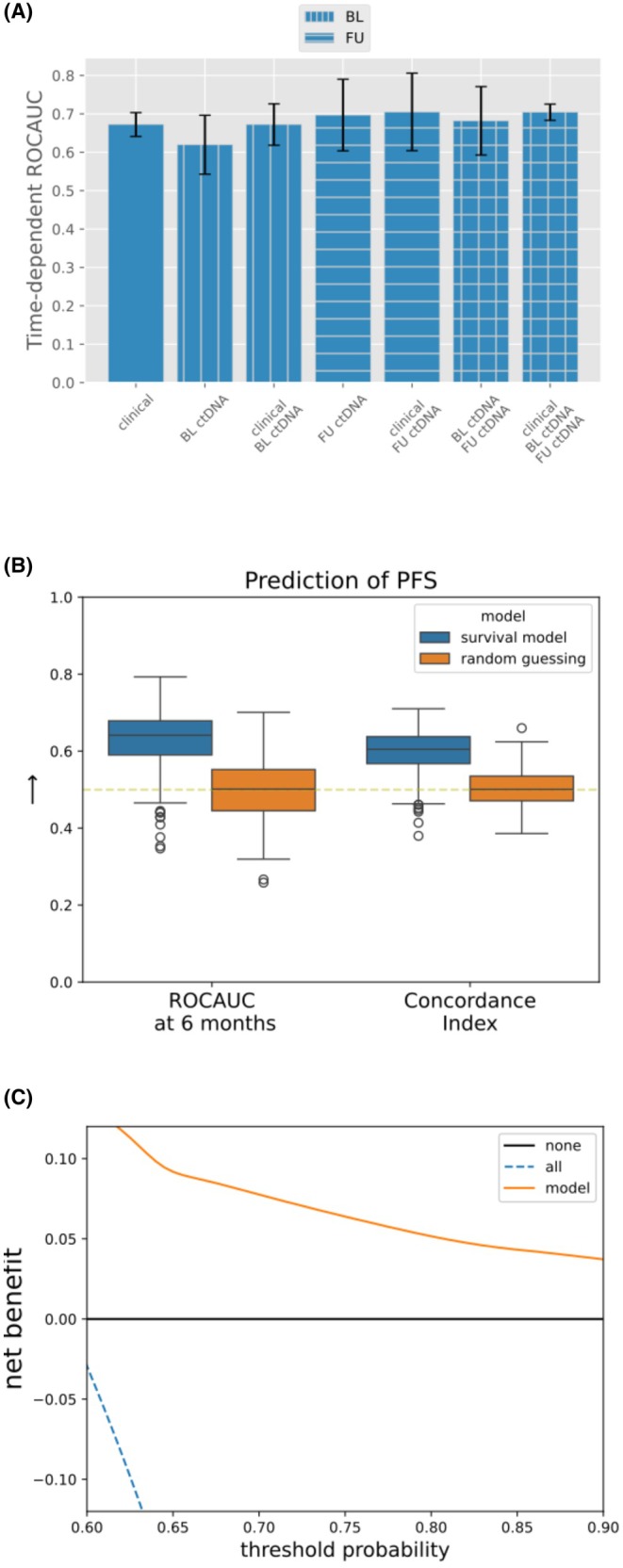
Progression‐Free Survival (PFS) prediction by machine learning. (A) ROCAUC (area under the receiver operator characteristic) at 6 months, (y‐axis) achieved by each different combination of features (x‐axis). The mean and standard error across 10 cross‐validation folds is shown for the best survival model and hyperparameter combination per feature combination. Combinations including baseline aneuploidy measurements (t_BL_)are designated with a vertical pattern and combinations including aneuploidy measurements at the on‐treatment timepoint (t_OT_) with a horizontal pattern. (B) Estimated performance of our prediction model (blue) and a random model (orange) on new unseen data from the same population. The distribution of the ROCAUC at 6 months and concordance index across 200 train‐test splits is shown on the y‐axis (higher is better for both). The median is denoted by a horizontal bar and boxes show the interquartile range (IQR), while the whiskers extend from the boxes to the farthest data point lying within 1.5 × the IQR. Points outside that range are shown as circles. (C) Decision curve analysis comparing the net benefit (y‐axis) of different strategies to guide clinical decisions for a wide range of clinically relevant probability thresholds (x‐axis) for 102 patients eligible for multivariable analysis. Our model's net benefit (orange line) is compared to that of intervene for none (black) and intervene for all (dashed blue).

The best‐performing model was a Cox proportional hazards model with elastic net regularization, while clinical data combined with ctDNA at t_OT_ were the most informative measurements (Fig. 3A). More specifically, the best model used the number of treatment lines and the continuous aneuploidy score at t_OT_, a finding that was robust to data resamplings (Fig. S4). The binarized aneuploidy score was not used, implying that the previously reported cutoff of 5 is suboptimal for the task at hand. The dynamics features were not used either, emphasizing the high importance of the t_OT_ sample for estimating the risk of progression.

The expected performance of this model on new patients is shown in Fig. 3B and compared to a model that is assigning a random risk to each patient. The median concordance index was 0.60 and was reliably higher than random. This means that for a pair of patients, the patient with more rapid progression is expected to be assigned a larger risk by the model 60% of the time. In terms of predicting progressive disease within 6 months from the initiation of pembrolizumab, the model's median ROCAUC was 0.64 and again clearly outperformed the random baseline (Fig. 3B). These results confirm the prognostic information present in the on‐treatment mFAST‐SeqS aneuploidy score.

Decision curve analysis showed that internally validated predictions of our model outperformed both the ‘intervene for none’ and ‘intervene for all’ strategies in terms of net benefit consistently and across the entire range of clinically relevant probability thresholds (Fig. 3C). Our model's predictions were also superior to those of PD‐L1 status, which—on its own—did not consistently outperform ‘intervene for none’ (Fig. S5). These results provide further evidence that our model can potentially be used for guiding clinical decision‐making in terms of identifying early on‐treatment a sub‐group of patients with poor prognosis who are unlikely to benefit from ICI treatment.

## 4 Discussion

In this study, we show that an increased mFast‐SeqS‐based aneuploidy score early during treatment with pembrolizumab is a robust marker for poor prognosis in mUC patients and could guide decisions on treatment (dis)continuation.

Previously, we have shown in mUC patients that a high aneuploidy score at baseline, prior to initiation of pembrolizumab, is strongly associated with poor prognosis [19]. However, a low aneuploidy score was not discriminative, meaning that some patients with a low aneuploidy score at baseline still lacked benefit from pembrolizumab treatment [19].

With an additional measurement of the mFast‐SeqS‐based aneuploidy score after the first or second cycle of pembrolizumab, we can discriminate patients with a poor prognosis and expected lack of benefit from pembrolizumab very early during treatment. In fact, this early on‐treatment measurement turned out to be more informative than the baseline measurement. Both baseline and on‐treatment high mFast‐SeqS based ctDNA amounts, as well as a lack of ctDNA decrease over time were strongly associated with poor overall survival, but the single baseline score was not significant in terms of PFS in multivariable analyses in this dataset.

Our findings are in line with the recently published exploratory data from the KEYNOTE‐361 trial. In this trial, pembrolizumab with and without chemotherapy was compared with chemotherapy alone in patients with mUC. The exploratory analysis showed a clear association between ctDNA dynamics and response and survival outcomes in patients treated with pembrolizumab. High or increasing amounts of ctDNA were related to worse outcomes [24]. However, our work shows for the first time how a relatively insensitive, but easily implementable assay can be used for this purpose. We additionally found evidence that dynamics might hold greater promise as a prognostic tool for patients receiving pembrolizumab in the second line, although these interaction analyses need to be interpreted with caution due to the relatively small number of patients treated with pembrolizumab in the first line in our cohort.

Biomarkers that can support clinicians with decision‐making on initiation or (dis)continuation of ICI‐based therapies in mUC patients are needed. Except for PD‐L1, there are no other reliable supportive biomarkers available in daily practice yet. ctDNA may provide a promising option, but the use of somatic mutations as biomarker requires either prior knowledge on the mutation status of a patient's tumor, large sequencing panels, or tumor‐informed patient‐specific panels, all of which complicate clinical implementation. The mFast‐SeqS method is a tumor‐naïve approach without the need for prior information on the molecular makeup of the tumor. Advantages include that it is relatively cheap (only 0.1 m sequencing reads needed), fast (~ 1.5 days with ~ 30 min hands‐on time), and only needs 0.5–1 ng of cfDNA input. With chromosomal instability being one of the general hallmarks of cancer, the mFast‐SeqS approach is a generally applicable method across tumor types. This greatly increases the feasibility for clinical implementation compared to tumor‐informed assays and could facilitate health care equity worldwide. Nevertheless, there are several limitations with respect to this approach. Technical sensitivity is low as it needs ctDNA levels around 5–10% to become positive [17]. However, our results suggest this might be sufficient to identify patients with poor prognosis and expected non‐response to pembrolizumab, as these patients typically have higher ctDNA levels.

Next to the limited technical sensitivity, another important limitation of the mFast‐SeqS is the use of a threshold to define samples as ctDNA high or low. This chosen threshold allows for the accurate detection of samples with a high ctDNA load, which was previously shown to be prognostic in real‐world metastatic cancer patients [25]. As demonstrated by our survival prediction experiment, the continuous aneuploidy scores proved to be more informative in this setting. However, also using the predefined cutoff of 5 on the on‐treatment samples or using the continuous ratio relative to baseline showed significant associations with clinical outcome and may be more easily implementable in clinical practice.

We tested the value of the mFast‐SeqS aneuploidy scores, that is, the ctDNA levels, in a machine learning‐based survival model. Although the used model's performance is still far from perfect, decision curve analysis confirmed that it can potentially be useful for identifying patients with a poor prognosis who are unlikely to benefit from continuing with pembrolizumab. Our results thus serve as a crucial first step toward a simple, affordable assay and a user‐friendly and automated tool based on clinical and ctDNA parameters that can help reduce the number of patients treated with pembrolizumab.

Based on the data from our study, we cannot discriminate whether the observed associations between the aneuploidy scores and clinical outcomes are prognostic irrespective of the treatment given, or predictive for pembrolizumab specifically. Acknowledging the rapidly changing treatment landscape in UC, we anticipate pembrolizumab monotherapy will not remain the mainstay for many patients in the near future, indicating that future work should investigate the value of these measurements in the changing treatment paradigm. Several recent studies in metastatic, adjuvant, or peri‐operative settings have shown the value of ctDNA as an important prognostic factor [26, 27, 28, 29, 30], which is paving the way for incorporating ctDNA in treatment decisions in patients with UC. Results with the same ctDNA assay in advanced breast cancer patients highlighted the relative benefit of immediate treatment escalation in these baseline ctDNA‐high patients [18]. Based on this, one could envision a similar scenario in metastatic UC, where baseline aneuploidy‐based ctDNA results could be used to decide which patients should start with an escalated treatment regimen (combination systemic therapy like ADC‐ICI) and which patients could start with a more conservative treatment strategy (ICI or ADC monotherapy). Subsequent on‐treatment ctDNA dynamics within this latter group could identify additional patients in need of treatment escalation. This ctDNA‐guided strategy should be compared to standard clinical care not only in terms of clinical outcome but also in terms of observed toxicity, quality of life, and costs.

## 5 Conclusion

Based on the results from this study, mFast‐SeqS based ctDNA levels and dynamics provide prognostic biomarkers that hold the promise to guide early clinical decision on initiation and (dis)continuation of PD1 checkpoint inhibition in patients with mUC, in a tumor‐agnostic, fast and affordable fashion. This may open up the possibility to shift to another treatment option in a timely fashion.

## Conflict of interest

*ItP:* Educational presentation at master class organized by Benecke—All paid to the Radboud university medical center. *JV:* consultancy and advisory board fees: J&J (to the institution), Leo Pharma (to the institution). *AAMvdV:* Consultancy fees (all paid to the institute): BMS, MSD, Sanofi, Regeneron, Ipsen, Novartis, GE Healthcare, Pierre Fabre. *RdW:* Speaker and advisory role: Astellas, advisory role: Merck US. *NM*: reports grants from Janssen‐Cilag, Astellas Pharma, Astrazeneca, Bristol Myers Squibb Foundation, MSD Oncology, personal fees for consulting or advisory role from Janssen‐Cilag, Astellas Pharma, AstraZeneca, MSD Oncology, Bayer and Pfizer. *DGJR:* consultancy and advisory board fees: Merck Ag (to the institution), Pfizer (to the institution), Astellas (to the institution), Astrazeneca (to the institution), Amgen (to the institution), J&J (to the institution) and grants: Merck Ag (study grant to the institution), MSD (study grant to the institution). The remaining authors have no conflicts of interest to declare.

## Author contributions

YS, SM, DGJR, and SMW were involved in conceptualization and methodology. YS and SM were involved in validation, data curation, visualization, writing – original draft and formal analysis. SM was involved in software. VdW, CB, and YS were involved in investigation. HMW, JV, MJBA, AAMvdV, MR, RdW, ItP, SvW, NM, and DGJR were involved in resources. DGJR and SMW were involved in supervision. YS and DGJR were involved in project administration. All authors were involved in writing – review and editing.

## Supporting information


**Fig. S1.** Distribution of log‐ratios of aneuploidy scores for technical replicates.
**Fig. S2.** Survival analyses based on baseline aneuploidy scores.
**Fig. S3.** Similarity of chromosome arm‐level Z‐scores between the two time points.
**Fig. S4.** Feature importances for survival regression.
**Fig. S5.** Decision curve for guiding treatment discontinuation.
**Table S1.** Staircase encoding for ordinal ctDNA dynamics model.
**Table S2.** Explanation of the seven features sets.
**Table S3.** Hyperparameters used for survival regression training.
**Table S4.** Effect of PD‐L1 combined positive score on aneuploidy scores.
**Table S5.** Effect of tumor mutational burden on aneuploidy scores.
**Table S6.** Effect of inclusion center on aneuploidy scores.
**Table S7.** Survival analyses using the continuous aneuploidy ratio.
**Table S8.** Survival analysis for response to pembrolizumab.
**Table S9.** Survival analyses including interactions between aneuploidy dynamics and treatment line.
**Table S10.** Survival analyses including the PD‐L1 combined positive score.

## Data Availability

Reasonable requests of the sequencing and clinical data used in this manuscript for research purposes should be addressed to the corresponding author (y.salhi@erasmusmc.nl).
