# Supplementary material for: Longitudinal genome‐wide aneuploidy measurements in circulating cell‐free DNA to predict lack of benefit from pembrolizumab in patients with metastatic urothelial cancer
Source: Mol Oncol. 2026 Jun 16:10.1002/1878-0261.70272. Online ahead of print. doi: 10.1002/1878-0261.70272 (PMC13398679; doi:10.1002/1878-0261.70272)
Supplement: Supplementary file 1 — Fig. S1. Distribution of log‐ratios of aneuploidy scores for technical replicates. Fig. S2. Survival analyses based on baseline aneuploidy scores. Fig. S3. Similarity of chromosome arm‐level Z‐scores between the two time points. Fig. S4. Feature importances for survival regression. Fig. S5. Decision curve for guiding treatment discontinuation. Table S1. Staircase encoding for ordinal ctDNA dynamics model. Table S2. Explanation of the seven features sets. Table S3. Hyperparameters used for survival regression training. Table S4. Effect of PD‐L1 combined positive score on aneuploidy scores. Table S5. Effect of tumor mutational burden on aneuploidy scores. Table S6. Effect of inclusion center on aneuploidy scores. Table S7. Survival analyses using the continuous aneuploidy ratio. Table S8. Survival analysis for response to pembrolizumab. Table S9. Survival analyses including interactions between aneuploidy dynamics and treatment line. Table S10. Survival analyses including the PD‐L1 combined positive score. [file MOL2-9999-0-s001.docx]

**Longitudinal genome-wide aneuploidy measurements in circulating cell-free DNA to predict for lack of response to pembrolizumab in patients with metastatic urothelial cancer**

Supplementary Material

**Supplementary figures**


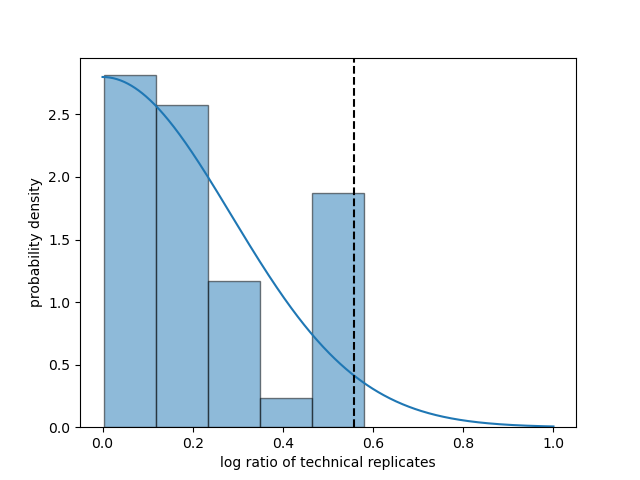


**Figure S1**: Distribution (histogram of the absolute log ratios or aneuploidy scores for 37 patients with breast cancer for which we generated two technical replicates. The blue curve shows the maximum likelihood fit of a half-normal distribution and the horizontal dashed line our cut-off.

| **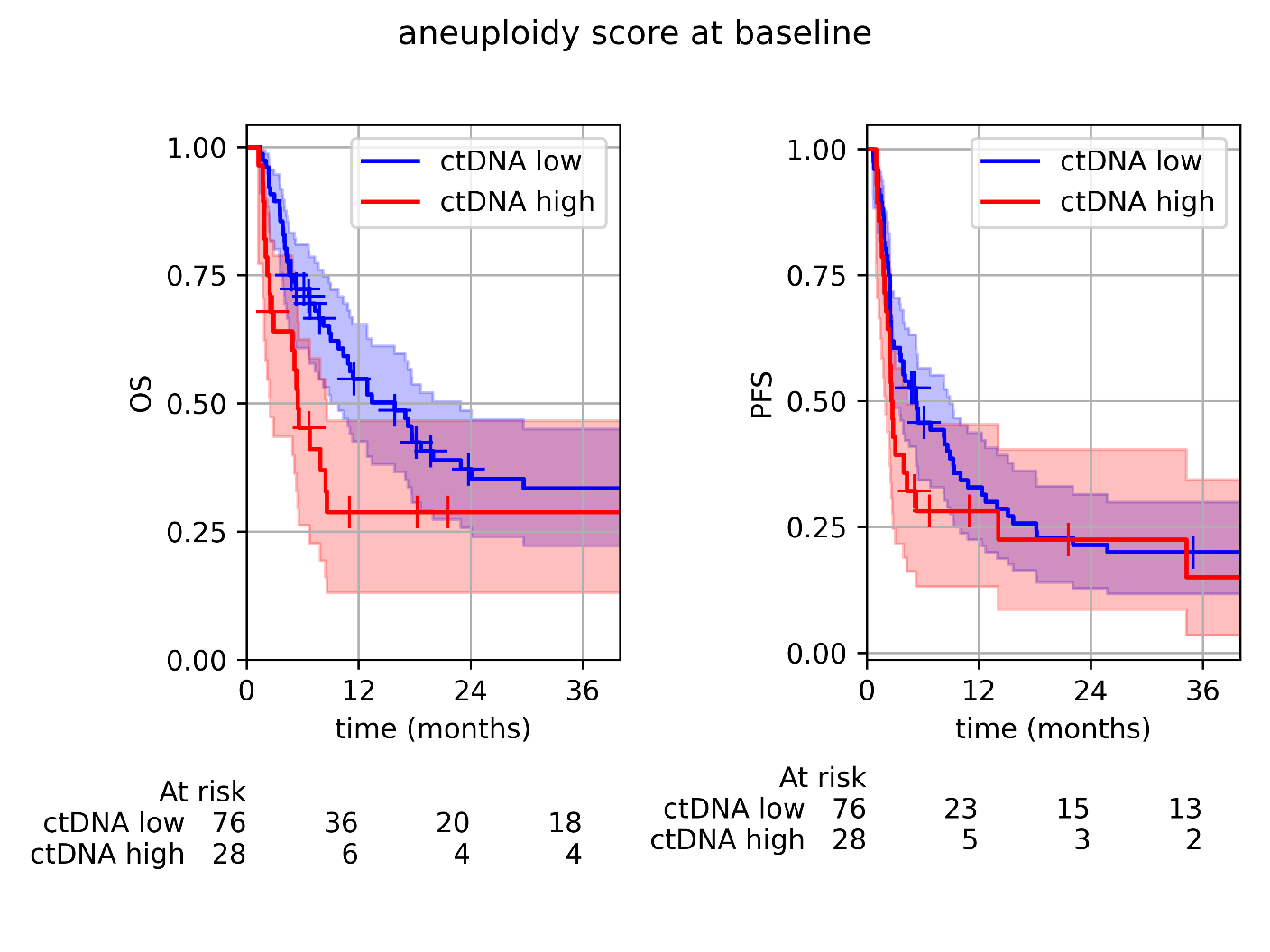** |
| --- |
| **Figure S2:** Kaplan-Meier curves for overall survival (left) and progression-free survival (right) of patients with a high (red) and low (blue) aneuploidy score at **t_BL_**. Shaded areas denote 95% confidence intervals. |


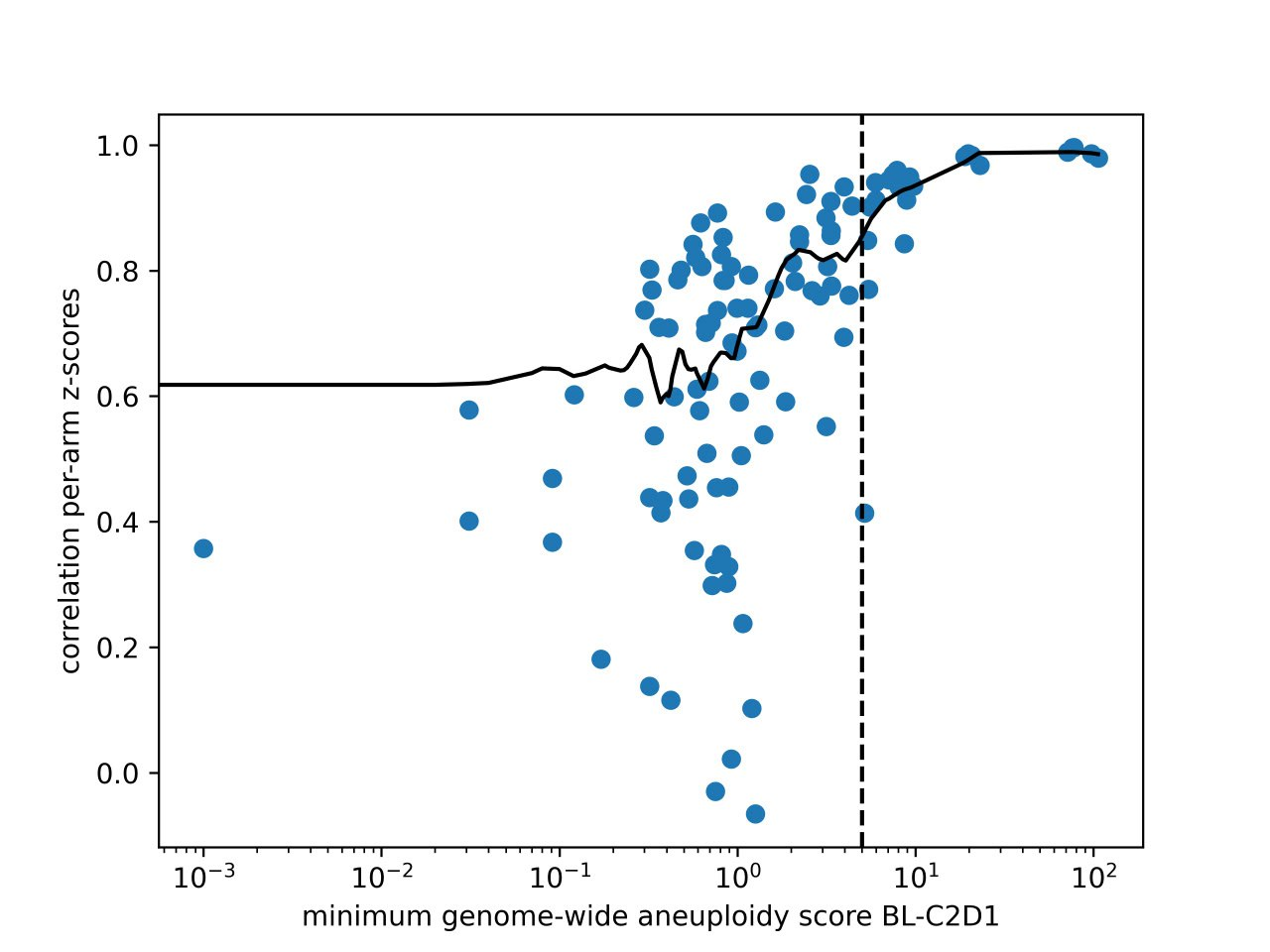


**Figure S3:** For each patient we evaluated the similarity of the pre-treatment and the on-treatment copy number profiles by calculating the Pearson correlation of the per-arm z-scores (y-axis). For ctDNA positive patients, these z-scores reflect the tumor-derived copy number changes and should be highly correlated over the two time points if the tumor’s copy number profile does not change. On the other hand, for ctDNA negative or low samples, it is not possible to assess the chromosomal aberrations and the z-scores can be more noisy. The correlations of the per arm z-scores are shown as a function of the minimum genome-wide aneuploidy score of each patient in the two time points (x-axis, in log scale). We found that the profiles were highly correlated, especially for samples with genome-wide z scores above 5 (vertical dashed line) with a lot of ctDNA. The sold black line shows the non-linear trend between the minimum aneuploidy score and the correlation estimated by loess regression.

| 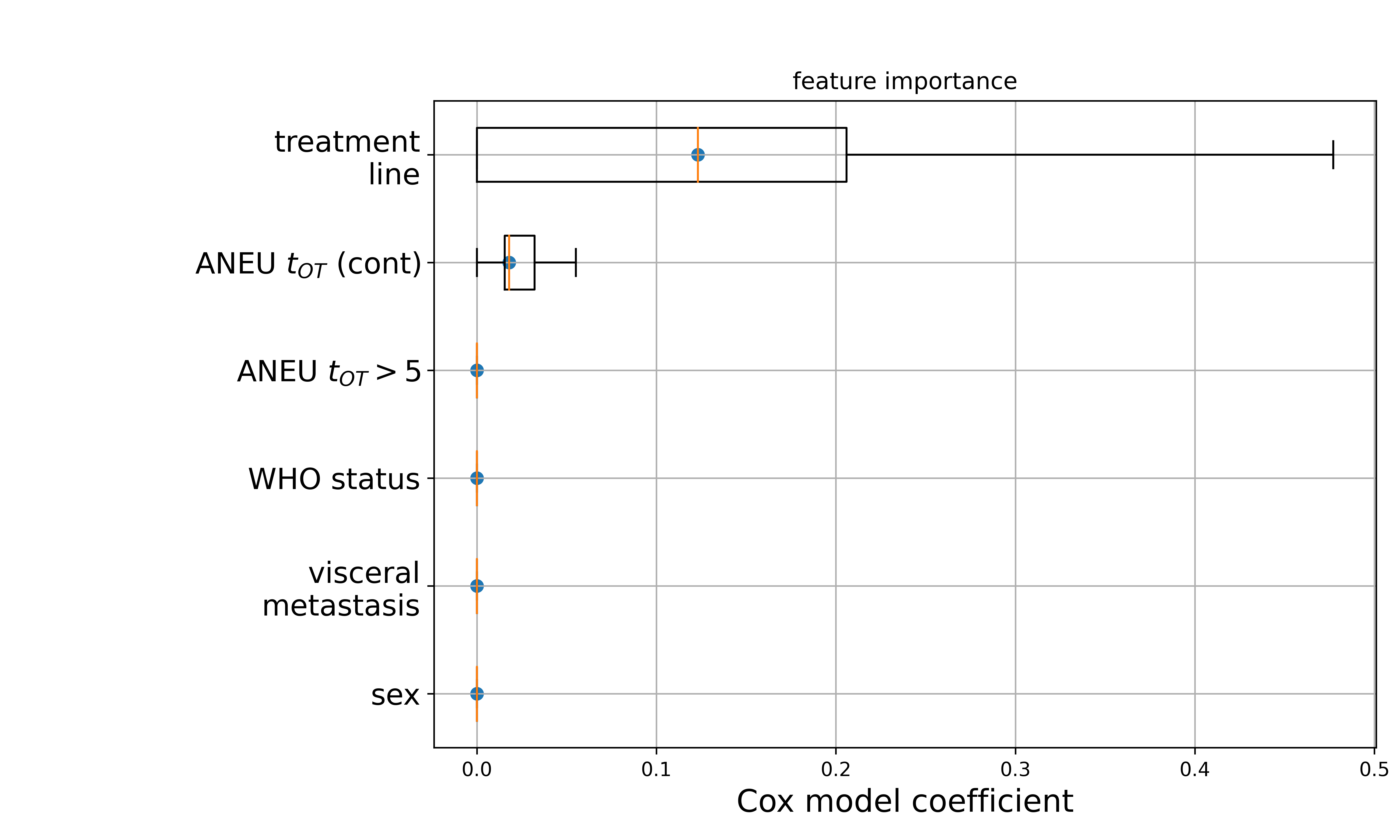 |
| --- |
| **Figure S4:** Feature importance (blue dots) measured by the weight (x-axis) each feature (y-axis) got assigned by the best model. The robustness of these feature weights was tested by performing 200 bootstraps, which are visualized using boxes. Per feature, the box shows the IQR, while the whiskers extend to 1.5 times the IQR away from the box. |

| 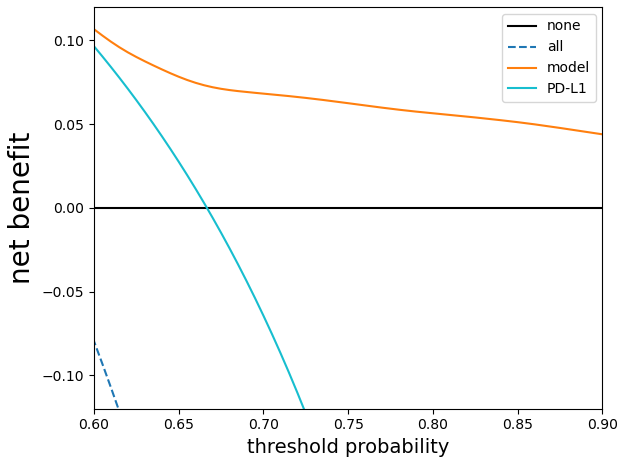 |
| --- |
| **Figure S5:** Decision curve analysis comparing the net benefit (y-axis) of different strategies to guide clinical decisions for a wide range of clinically relevant probability thresholds (x-axis) for 88 patients with known PD-L1 status. Our model’s net benefit (orange line) is compared to that of PD-L1 (cyan), intervene for none (black) and intervene for all (dashed blue). |

**Supplementary tables**

| **Table S1:** Illustration of how the ctDNA dynamics groups were encoded into an ordinal variable. |
| --- |
| \| **Group\Variable** \| **ctDNA stable vs decrease** \| **ctDNA increase vs stable** \| \| --- \| --- \| --- \| \| **ctDNA-decrease** \| 0 \| 0 \| \| **ctDNA-stable** \| 1 \| 0 \| \| **ctDNA-increase** \| 1 \| 1 \| |

**Table S2:** The features used for our prediction models (rows) categorized into feature sets (columns). A cell marked by ‘x’ denotes that the corresponding feature was included in the corresponding set. (n = 101)

| **Variable\feature set** | **1**  **clinical** | **2**  **t_BL_** | **3**  **clinical/**  **t_BL_** | **4**  **t_OT_** | **5**  **clinical/**  **t_OT_** | **6**  **t_BL_ / t_OT_** | **7**  **clinical/**  **t_BL_ / t_OT_** |
| --- | --- | --- | --- | --- | --- | --- | --- |
| Sex | **X** |  | **x** |  | **x** |  | **x** |
| Visceral metastasis | **X** |  | **x** |  | **x** |  | **x** |
| WHO status | **X** |  | **x** |  | **x** |  | **x** |
| Aneuploidy score  **t_BL_** (continuous) |  | **x** | **x** |  |  | **x** | **x** |
| Aneuploidy score  **t_BL_** ≥ 5 |  | **x** | **x** |  |  | **x** | **x** |
| Aneuploidy score  **t_OT_** (continuous) |  |  |  | **x** | **x** | **x** | **x** |
| Aneuploidy score  **t_OT_** ≥ 5 |  |  |  | **x** | **x** | **x** | **x** |
| Aneuploidy stable |  |  |  |  |  | **x** | **x** |
| Aneuploidy increase |  |  |  |  |  | **x** | **x** |

**Table S3:** Survival predictors, their corresponding hyperparameters and for each hyperparameter, the values that were considered in our grid search.

| **classifier** |  |  |  |  |
| --- | --- | --- | --- | --- |
| survival SVM | **regularization strength** | **rank ratio** |  |  |
|  | [0.01, 0.1, 0.2, 0.5, 1.0, 2.0, 5.0, 10.0, 20.0, 50.0, 100.0] | [0.0, 0.1, 0.2, 0.3, 0.4, 0.5, 0.6, 0.7, 0.8, 0.9, 1.0 |  |  |
| Cox regression | **regularization strength** | **L1 ratio** |  |  |
|  | [0.0001, 0.0005, 0.001, 0.005, 0.01, 0.05, 0.1, 0.2, 0.5, 1.0, 2.0, 5.0, 10.0] | [1e-5, 0.1, 0.2, 0.3, 0.4, 0.5, 0.6, 0.7, 0.8, 0.9, 1.0] |  |  |
| Survival forest | **number of trees** | **min sample size for split** | **Maximum tree depth** | **Maximum features**  **per tree** |
|  | [10, 20, 50, 100, 200,400] | [4,6,8] | [1,2,3] | [sqrt(N), log(N)] |
| Survival tree | **min sample size for split** | **Maximum tree depth** |  |  |
|  | [4,6,8] | [2,3,5,10] |  |  |

| **Table S4:** Results of a mixed effects model that models log-transformed aneuploidy scores based on time point and PD-L1. Statistically significant variables are shown in bold. The model was fitted on 178 observations from 89 individuals. |
| --- |
| \|  \| coefficient \| standard error \| 95% CI \| p-value \| \| --- \| --- \| --- \| --- \| --- \| \| Intercept \| 1.50 \| 0.16 \| [1.19, 1.80] \| <10^-3^ \| \| **timepoint**  **(ref t_BL_)** \| **-0.23** \| **0.09** \| **[-0.40, -0.06]** \| **0.008** \| \| PD-L1 CPS≥10 \| -0.36 \| 0.23 \| [-0.81, 0.10] \| 0.13 \| \| Group variance \| 1.01 \| 0.43 \|  \|  \| |

| **Table S5:** Results of a mixed effects model that models log-transformed aneuploidy scores based on time point and tumor mutational burden. Statistically significant variables are shown in bold. The model was fitted on 132 observations from 66 individuals. |
| --- |
| \|  \| coefficient \| standard error \| 95% CI \| p-value \| \| --- \| --- \| --- \| --- \| --- \| \| Intercept \| 1.68 \| 0.20 \| [1.28, 2.07] \| <10^-3^ \| \| timepoint  (ref t_BL_) \| -0.20 \| 0.10 \| [-0.40, 0.01] \| 0.06 \| \| TMB ≥ 10 \| -0.44 \| 0.29 \| [-1.00, 0.12] \| 0.12 \| \| Group variance \| 1.15 \| 0.55 \|  \|  \| |

| **Table S6:** Results of a mixed effects model that models log-transformed aneuploidy scores based on time point, cohort, number of treatment lines, and WHO status. Statistically significant variables are shown in bold. The model was fitted on 206 observations from 103 individuals. |
| --- |
| \|  \| coefficient \| std error \| 95% CI \| p-value \| \| --- \| --- \| --- \| --- \| --- \| \| **intercept** \| **1.31** \| **0.34** \| **[0.61, 1.98]** \| **<0.001** \| \| **timepoint**  (ref. t_BL_) \| **-0.19** \| **0.08** \| **[-0.34, -0.04]** \| **0.013** \| \| cohort  (ref. PRECISE) \| -0.14 \| 0.23 \| [-0.59, 0.32] \| 0.55 \| \| pembrolizumab in 2nd line \| 0.19 \| 0.25 \| [-0.29, 0.67] \| 0.44 \| \| WHO status > 0 \| -0.06 \| 0.28 \| [-0.61, 0.50] \| 0.85 \| \| group variance \| 1.04 \| 0.43 \|  \|  \| |

**Table S7:** Multivariate analyses for prognostic value of the baseline aneuploidy score and its dynamics (aneuploidy ratio) as a continuous variable in terms of OS (left) and PFS (right). The hazard ratio (HR), 95% confidence interval around the HR estimate (CI), and the associated p-value are shown for each variable. The presence/absence or not of visceral metastasis violated the proportional hazards assumption (i.e. demonstrated a time-dependent hazard ratio), so we included it as a random effect and thus did not estimate its hazard (n = 102).

|  | OS | | | PFS | | |
| --- | --- | --- | --- | --- | --- | --- |
|  | **HR** | **95% CI** | **p-value** | **HR** | **95% CI** | **p-value** |
| pembrolizumab  in 2^nd^ line | 2.34 | [1.26, 4.36] | 0.007 | 3.64 | [1.97, 6.74] | <0.001 |
| Sex  (ref. Female) | 0.65 | [0.37, 1.15] | 0.15 | 0.58 | [0.34, 0.99] | 0.045 |
| Study  (ref. PRECISE) | 2.06 | [1.19, 3.59] | 0.01 | 1.31 | [0.79, 2.15] | 0.30 |
| WHO status > 0 | 1.74 | [0.87, 3.51] | 0.12 | 1.28 | [0.70, 2.34] | 0.42 |
| Aneuploidy ≥ 5 at tBL | 2.33 | [1.27, 4.24] | 0.005 | 1.63 | [0.93, 2.84] | 0.09 |
| ctDNA dynamics (continuous) | 1.48 | [1.19, 1.85] | <0.001 | 1.38 | [1.11, 1.70] | 0.003 |

| **Table S8:** Multivariable analyses for prognostic value of the baseline aneuploidy score and its dynamics over the first treatment course in terms of response to ICI. Response was defined as stable disease, partial response, or complete response after 6 months. Association was tested using a Cox proportional hazards model for PFS where all samples were censored at 6 months (type I censoring). The hazard ratio (HR), 95% confidence interval around the HR estimate (CI), and the associated p-value are shown for each variable. The presence/absence of visceral metastasis violated the proportional hazards assumption (i.e. demonstrated a time-dependent hazard ratio), so we included it as a random effect and did not estimate its hazard). |
| --- |
| \|  \| **HR** \| **95% CI** \| **p-value** \| \| --- \| --- \| --- \| --- \| \| **Pembrolizumab in 2nd line** \| **2.63** \| **[1.33, 5.18]** \| **0.01** \| \| sex \| 0.59 \| [0.32, 1.06] \| 0.08 \| \| Study \| 1.46 \| [0.83, 2.58] \| 0.19 \| \| WHO >0 \| 1.64 \| [0.77, 3.51] \| 0.20 \| \| Aneuploidy >5 BL \| 1.78 \| [0.93, 3.41] \| 0.08 \| \| **ctDNA-stable vs ctDNA-decrease** \| **2.17** \| **[1.08, 4.38]** \| **0.03** \| \| ctDNA-increase vs ctDNA-stable \| 1.76 \| [0.74, 4.21] \| 0.20 \| |

| **Table S9:** Multivariable analyses of OS and PFS including an interaction term between aneuploidy score dynamics (continuous) and treatment line (n=102). The row ‘aneuploidy dynamics’ corresponds to the effect of the dynamics in first-line patients and the interaction term ‘pembrolizumab in 2^nd^ line × aneuploidy dynamics’ corresponds to the effect in second-line patients. |
| --- |
| \|  \| **OS** \| \| \| **PFS** \| \| \| \| --- \| --- \| --- \| --- \| --- \| --- \| --- \| \|  \| **HR** \| **95% CI** \| **p-value** \| **HR** \| **95% CI** \| **p-value** \| \| age \| 1.01 \| [0.98, 1.05] \| 0.50 \| 1.01 \| [0.98, 1.03] \| 0.72 \| \| cohort \| **2.04** \| **[1.18, 3.56]** \| **0.01** \| 1.30 \| [0.79, 2.12] \| 0.30 \| \| sex \| 0.65 \| [0.37, 1.15] \| 0.14 \| 0.60 \| [0.35, 1.04] \| 0.07 \| \| WHO status \| 1.75 \| [0.86, 3.57] \| 0.12 \| 1.27 \| [0.69, 2.36] \| 0.44 \| \| aneuploidy **t_BL_** \| **2.35** \| **[1.28, 4.29]** \| **0.01** \| 1.58 \| [0.90, 2.78] \| 0.11 \| \| pembrolizumab in 2^nd^ line \| **2.44** \| **[1.30, 4.59]** \| **0.01** \| **3.88** \| **[2.09, 7.19]** \| **0.001** \| \| aneuploidy dynamics \| 1.37 \| [0.99, 1.91] \| 0.06 \| 1.04 \| [0.69, 1.56] \| 0.86 \| \| pembrolizumab in 2^nd^ line ×  aneuploidy dynamics \| 1.01 \| [0.98, 1.05] \| 0.50 \| **1.68** \| **[1.02, 2.78]** \| **0.04** \| |

| **Table S10:** Multivariate analyses for prognostic value of the baseline aneuploidy score and its dynamics over the first treatment course in terms of OS (left) and PFS (right) for the subset of patients (n=88) for whom PD-L1 status was assessed. The hazard ratio (HR), 95% confidence interval around the HR estimate (CI), and the associated p-value are shown for each variable. The presence/absence of visceral metastasis violated the proportional hazards assumption (i.e. demonstrated a time-dependent hazard ratio), so we included it as a random effect and thus did not estimate its hazard. (n = 102) |
| --- |
| \|  \| **OS** \| \| \| **PFS** \| \| \| \| --- \| --- \| --- \| --- \| --- \| --- \| --- \| \|  \| **HR** \| **CI** \| **p-value** \| **HR** \| **CI** \| **p-value** \| \| Pembrolizumab in 2^nd^ line \| 1.87 \| [0.84, 4.16] \| 0.12 \| 3.22 \| [1.55-6.67] \| 0.002 \| \| **Sex (ref. Female)** \| 0.59 \| [0.32, 1.10] \| 0.10 \| 0.46 \| [0.25-0.85] \| 0.013 \| \| **Study (ref. PRECISE)** \| 3.12 \| [1.50-6.45] \| 0.002 \| 2.44 \| [1.29-4.62] \| 0.006 \| \| **WHO status > 0** \| 2.21 \| [1.05-4.66] \| 0.037 \| 1.69 \| [0.89-3.22] \| 0.11 \| \| **PD-L1 CPS > 10** \| 0.46 \| [0.23-0.92] \| 0.029 \| 0.49 \| [0.22-0.73] \| 0.003 \| \| **Aneuploidy ≥ 5 at tBL** \| 2.22 \| [1.11-4.42] \| 0.024 \| 1.71 \| [0.90-3.23] \| 0.10 \| \| **ctDNA stable vs decrease** \| 2.42 \| [1.24-4.73] \| 0.01 \| 2.27 \| [1.23-4.18] \| 0.009 \| \| **ctDNA increase vs stable** \| 0.99 \| [0.38-2.58] \| 0.98 \| 1.20 \| [0.48-3.02] \| 0.70 \| |
